# Iatrogenic Cerebral Amyloid Angiopathy in Patients Treated With Cadaveric Dura Mater During Childhood Neurosurgery: A Retrospective Cohort Study

**DOI:** 10.1111/ene.70091

**Published:** 2025-03-06

**Authors:** Slaven Pikija, Andreea Toma, Richard Radlberger, Christoph J. Griessenauer, Constantin Hecker, Eugen Trinka, Park Hyon Ki, Theo Kraus, Serge Weis, Marieke J. H. Wermer, Ellis S. van Etten, Kanishk Kaushik

**Affiliations:** ^1^ Department of Neurology, Neurocritical Care and Neurorehabilitation, Member of the European Reference Network EpiCARE, Christian Doppler University Hospital Salzburg Paracelsus Medical University Salzburg Austria; ^2^ Neuroscience Institute, Christian Doppler University Hospital Salzburg Centre for Cognitive Neuroscience Paracelsus Medical University Salzburg Austria; ^3^ Department of Neurosurgery, Christian Doppler University Hospital Salzburg Paracelsus Medical University Salzburg Austria; ^4^ Institute of Neurointervention Paracelus Medical University Salzburg Austria; ^5^ Department of Public Health, Health Services Research and Health Technology Assessment, UMIT – University for Health Sciences Medical Informatics and Technology Hall in Tirol Austria; ^6^ Department of Pathology General University Hospital Salzburg Salzburg Austria; ^7^ Department of Pathology Keppler University Centre Linz Austria; ^8^ Department of Neurology University Medical Center Groningen (UMCG) the Netherlands; ^9^ Department of Neurology Leiden University Medical Centre (LUMC) the Netherlands

**Keywords:** amyloid‐beta, cadaveric dura mater, iatrogenic cerebral amyloid angiopathy, iCAA, Lyodura, neurosurgery, pediatric, retrospective cohort studies

## Abstract

**Background:**

Iatrogenic cerebral amyloid angiopathy (iCAA) is a recently identified clinico‐neuroradiological syndrome associated with medical procedures, particularly neurosurgical treatments involving cadaveric dura mater (e.g., Lyodura). iCAA can manifest as intracerebral hemorrhages, focal seizures, and cognitive impairment, with the risk following exposure currently unknown. We aim to evaluate the risk of developing iCAA in patients who underwent childhood neurosurgical treatment with Lyodura compared to those who did not.

**Methods:**

This retrospective cohort study analyzed hospital records from the Christian‐Doppler University Hospital in Salzburg, along with mortality data provided by the Austrian Federal Institute of Statistics (Statistik Austria). The study included all patients aged 0–18 who underwent neurosurgical procedures between January 1970 and January 1996. The primary endpoint was the diagnosis of iCAA and iCAA‐related death.

**Results:**

Of 569 pediatric neurosurgical patients, 388 (68%) were further analyzed. Four patients (1%) were diagnosed with probable iCAA at a median age of 42 years (IQR 40–47), with a median latency from surgery to symptom onset of 38 years (IQR 36–41). Only Lyodura recipients developed iCAA, with an incidence rate of 12% (OR 56, 95% CI: 6–2667). The overall incidence of symptomatic iCAA among recipients of any dura material was 5% (OR 19, 95% CI: 2–903).

**Conclusions:**

Cadaveric dura mater, especially Lyodura, poses a long‐term risk for developing iCAA. Further research is needed to determine susceptibility factors in Lyodura‐exposed individuals.

## 1 Introduction

Iatrogenic cerebral amyloid angiopathy (iCAA) is a recently recognized syndrome that causes intracerebral hemorrhage (ICH) and cognitive decline. Cerebral amyloid angiopathy (CAA) is thought to be caused by the accumulation of vascular amyloid‐ß (Aß), which in iCAA is thought to be exogenous, introduced by medical procedures [1].

A key prerequisite in the recently proposed criteria for diagnosing probable and possible iCAA is relevant exposure to cadaveric human central nervous system tissues [2]. Similar to the transmission of iatrogenic Creutzfeldt‐Jakob disease (iCJD), exposures include procedures involving the brain, meninges, pituitary‐derived hormones, or surgical interventions involving the brain, spinal cord, or posterior eye [3, 4, 5].

A substantial number (39%) of the published iCAA patients received cadaveric dura mater grafts, specifically Lyodura (B. Braun Melsungen AG, Melsungen, Germany), frequently due to traumatic brain injury (TBI) or surgery for a brain tumor in childhood [2, 6, 7]. Given the decades‐long latency period, patients with iCAA are emerging in modern times [6, 7]. Pathological Aß proteins are thought to spread from the graft into surrounding brain tissue, leading to pathological Aß depositions years after exposure. However, the exact pathophysiological cascade remains unclear [8]. Approximately 90 cases of iCAA have been reported to date [2, 6, 7].

Because documentation regarding neurosurgery in the era when cadaveric dura was used is often missing, the risk of developing iCAA after exposure to neurosurgery with or without cadaveric dura mater remains unknown. This study aims to evaluate the long‐term risk of developing iCAA in pediatric patients who underwent neurosurgery between 1970 and 1996, based on a comprehensive health record system.

## 2 Methods

### 2.1 Study Population

This retrospective cohort study included all patients recorded in paper‐based hospital records at the neurosurgery and neurology departments, aged 0–18 (inclusive), admitted to the neurosurgical department at the Christian‐Doppler‐University Hospital, Salzburg, Austria, between January 1st, 1970, to January 1st, 1996 (both inclusive).

Patients were excluded if they lacked neurosurgical intervention or died during admission for neurosurgery. This study received local ethical committee approval (Land Salzburg Ethikkommission, approval Nr: 1131/2023). The study was conducted in accordance with the Strengthening the Reporting of Observational Studies in Epidemiology (STROBE) guidelines.

### 2.2 Data Collection

Paper records from the 1970s to 1990s were all archived separately at the neurosurgical department as part of routine care. The surgery reports are generally detailed and typically include all relevant clinical information (reason for admission, outcome), particularly the type of dura substitute material used. Between 1970 and 1996, pediatric patients were primarily operated at our clinic. However, due to limited resources, the operating team occasionally operated at nearby surgical facilities, particularly in the late 1980s. For all patients, data on the cause of death was obtained from the national population registry (“Bundesanstalt Statistik Österreich”), which recorded causes of death using ICD‐9 or ICD‐10 codes for all cases from 1970 to 2023 (see Supporting Information). Patients were linked to our hospital database using demographic information. We identified 61 out of 388 patients (16%) with available hospital records, and the data for these 61 patients is reported separately. Although we considered contacting all operated cases to assess the potential development of iCAA through clinical questionnaires and neuroimaging, this was not feasible due to resource limitations and ethical constraints regarding screening asymptomatic individuals for (iatrogenic) CAA. We acknowledge that asymptomatic iCAA cannot be excluded with certainty in cases with digital follow‐up data or in other parts of the cohort with only mortality data available. Therefore, in this paper, we consider iCAA as symptomatic iCAA and, for brevity, use the term ‘iCAA’ throughout the text (except in the abstract). Two living patients with symptomatic iCAA provided written informed consent for publication.

### 2.3 Study Objectives

The primary objective was to assess whether patients who received cadaveric dura transplants during pediatric neurosurgery developed iatrogenic cerebral amyloid angiopathy in adulthood. Specifically, the study tested whether the risk of developing iCAA is higher among patients treated with documented cadaveric dura transplants compared to those receiving neurosurgical treatment alone or with dura replacement of unknown material. Historical data suggest the concomitant use of various materials such as autologous fascia lata, collagen sponges combined with Vicryl, bovine pericardium (1970–1980), polyester (1980s), and Dacron material [9, 10]. However, none of these other materials were explicitly mentioned in the available surgical records, and we do not have an estimate of their possible usage frequency in our population.

### 2.4 Endpoints and Exposures

The primary endpoint was the diagnosis of probable or possible iCAA (a combined endpoint) according to published criteria [2], ascertained from available hospital records, or iCAA‐related death (defined as death due to complications of iCAA). The date of iCAA diagnosis was used as the endpoint date. For the diagnosis of CAA and stroke, we applied liberal assumptions while acknowledging that we could not definitively confirm the absence of sporadic CAA or stroke in the cohort, as asymptomatic cases cannot be excluded with certainty. Our assumptions were as follows: In the subset of the cohort with available neuroimaging, patients without evidence of CAA or stroke on imaging were labeled as ‘CAA‐negative’ and ‘stroke‐negative,’ respectively. For the remaining individuals, we only had mortality data or limited clinical information. In these cases, we assumed no occurrence of CAA or stroke if no causes of death consistent with either condition (e.g., lobar intracerebral hemorrhage, subarachnoid hemorrhage, or ischemic stroke) were documented, but rather found only unrelated causes (such as cardiac failure, pneumonia, or injury). For any living patients in this subgroup, we similarly concluded no CAA or stroke where there were no neurological or related conditions documented in our hospital's records during the study period. Censoring events included death and end of study. The cause of death was extracted from hospital records and from the national population registry. No autopsy information was recorded. We considered three exposure groups: those with neurosurgical intervention, those who received cadaveric dura mater (exclusively Lyodura), and those who received non‐specified dura substitute material.

### 2.5 Statistical Methodology and Analysis

#### 2.5.1 Descriptive Analysis

For descriptive analyses, cases were grouped by exposure type (cadaveric dura recipient vs. non‐recipient). Patient age at exposure, sex, year of operation, reason for hospital admission, latency time (when available), other diagnoses during follow‐up, death, age at death, cause of death, duration of follow‐up, occurrence of stroke in follow‐up, grading of CAA according to Boston Criteria 2.0 and Edinburgh criteria, and grading of iCAA according to proposed diagnostic criteria for iCAA were tabulated. Counts and proportions were provided for categorical variables, while medians and interquartile ranges were calculated for numerical values. Odds ratios were calculated with continuity correction (adding value of 1 in case of non‐positive cells) and tested with Fisher's exact test.

We calculated odds ratios, comparing iCAA development in patients who received some form of dura material to those who did not. Similarly, we compared the odds of patients who received cadaveric dura with patients who received dura of unknown type. In a secondary analysis, to provide reference odds from a negative control group, we assessed the number of patients who developed ischemic stroke in each of the exposure groups and calculated odds ratios accordingly. We employed Kaplan–Meier survival analysis to estimate the time to iCAA diagnosis between recipients of Lyodura and those without. The censor variable was death during the study period or the study period ending without iCAA diagnosis. The primary outcome was time free of iCAA diagnosis. Due to the zero occurrence in non‐recipients, the Cox regression and competing risk analysis (with death as a competing risk) could not be performed. R Software version 4.2.2 was used for all statistical analyses.

#### 2.5.2 Data Sharing Statement

Anonymized data will be shared with fellow researchers upon reasonable request.

## 3 Results

**FIGURE 1:**
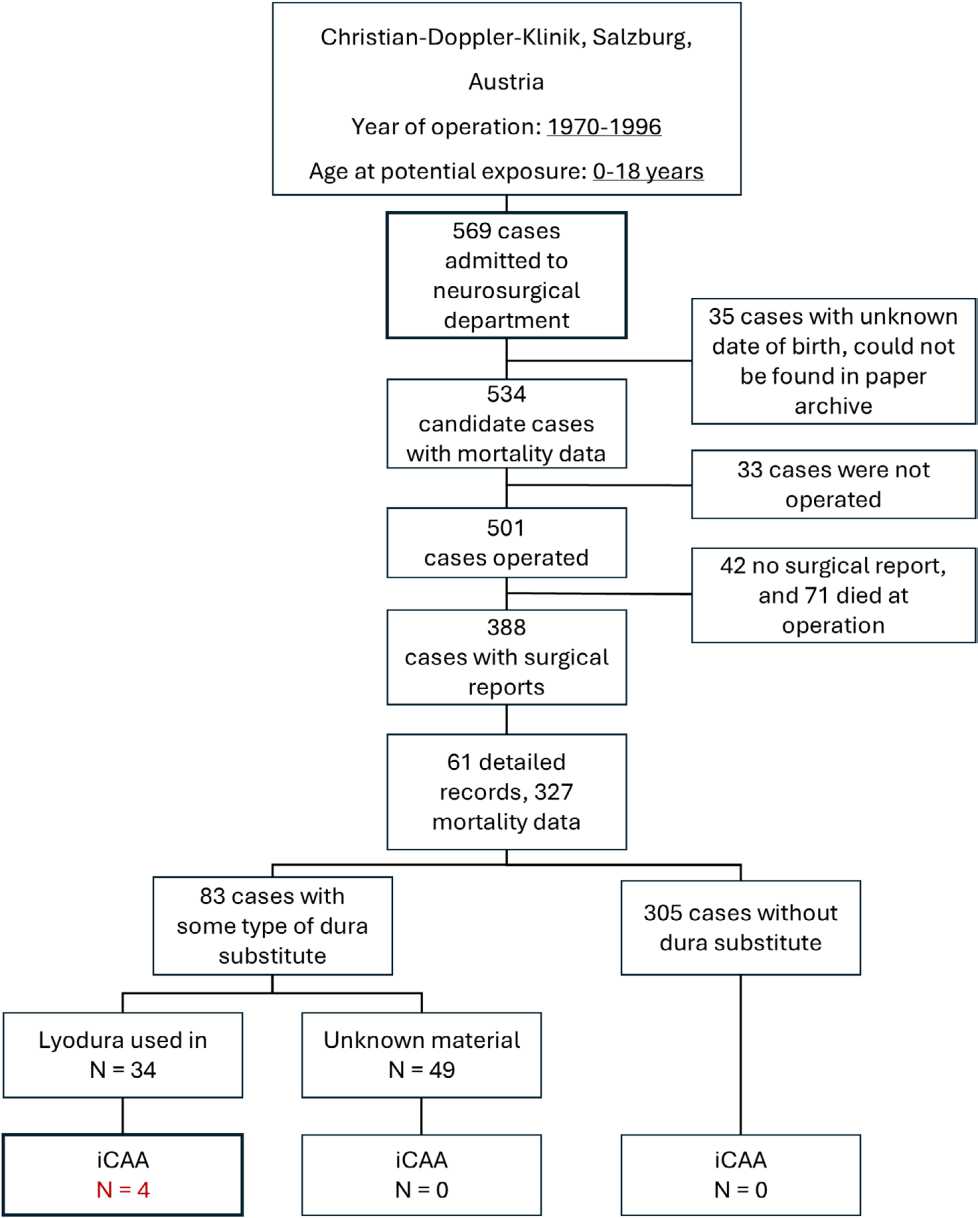
Patient flow chart.

A total of 569 records were identified as potential neurosurgical pediatric patients operated on between 1970 and 1996. Of these, 35/569 (6%) did not have an exact date of birth available. Death and its causes were ascertained for 534 patients through Statistik Österreich, with 129/534 (24%) deaths in the entire cohort. Ultimately, 33/534 (6%) patients did not undergo surgery, and for 42/501 (8%), the operation report could not be found. Among the 459 patients who had surgery, 71 (25%) died during the operation. As a result, 388/569 (68%) cases were available for further analysis (Figure 1), of whom 61 (16%) had records in the hospital database and 327 (84%) were supplemented by the national population registry. Here we report data on the entire cohort. The results and estimates for the 61 patients with available hospital medical records (beyond date, outcome, and causes) during the follow‐up are provided in the Supporting Information and Table S1.

The median age at the time of operation was 12.5 years (IQR 7–17 years). Of these patients, 69% were male (Table 1). Dura substitute was used in 83 cases (21%; 95 CI: 17%–26%), and Lyodura was explicitly mentioned in 34 patients (9%; 95% CI: 6%–12%). Out of 388 patients, 45/388 (12%) deaths occurred. The median follow‐up for the cohort was 40 years (IQR 36–46 years). No patients developed CJD.

**TABLE 1:** Demographic data on pediatric patients treated with neurosurgical procedures at the Christian‐Doppler‐Klinik, Salzburg, Austria, period 1970–1996.

Characteristic	*N* = 388
Men	268 (69)
Age at operation	12 (7–17)
Diagnosis at surgical admission	
TBI	278 (72)
Intracranial tumor	46 (12)
Other	42 (11)
Intracranial hemorhage	11 (2.8)
Intracranial abscess	7 (1.8)
Subarachnoidal bleeding	3 (0.8)
Unknown	1 (0.3)
Year of operation	1983 (1976–1986)
Type of dura material used	
None	305 (79)
Unknown material	49 (13)
Lyodura	34 (8.8)
Years of follow‐up	40 (36–46)
Died during follow‐up	45 (12)
Age at death (years)	30 (18–36)
Cause of death	
Other	8 (18)
Intracranial tumor	7 (16)
TBI	6 (13)
Trauma other than TBI	5 (11)
Unknown	4 (8.9)
Intracranial hemorhage	3 (6.7)
Cancer	3 (6.7)
Decompensatio cordis/myocardial infarction	4 (8.8)
Intracranial abscess	1 (2.2)
Hydrocephalus	1 (2.2)
Chronic lung disease	1 (2.2)
Pneumonia	1 (2.2)
Sepsis	1 (2.2)
CT done	33 (8.5)
MRI done	20 (5.2)
Diagnosis of iatrogenic CAA	
Not reported	327 (84)
Negative	57 (15)
Yes	4 (1.0)
Diagnosis of stroke	4 (1)
^1^ *n* (%); Median (IQR)	

*Note:* Other causes of death (*N* = 8[18%]): epileptic seizures 4 (50%), carbon monoxide poisoning 1 (12%), “other paralytic syndromes” 1 (12%), disorders involving the immune mechanism 1 (12%), and other diseases of intestine 1 (12%).

Abbreviations: CAA, cerebral amyloid angiopathy; CT, computerized tomography of brain; MRI, magnetic resonance imaging of brain; TBI, traumatic brain injury.

**FIGURE 2:**
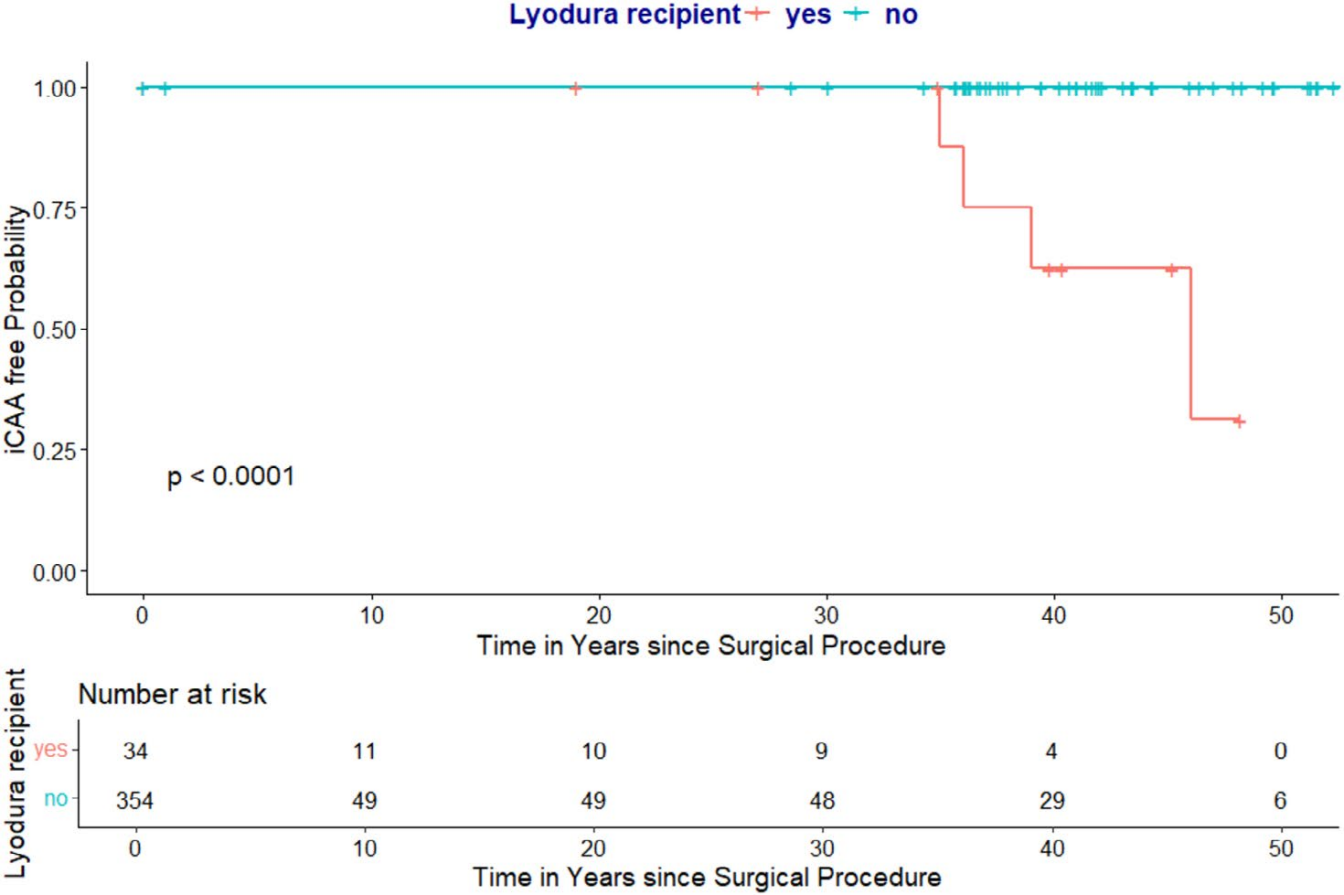
Kaplan–Meier curves representing a cumulative incidence of iatrogenic cerebral amyloid angiopathy over time after neurosurgery in pediatric age, stratified by the recipient of Lyodura.

We identified four cases (1%; 95% CI: 0%–3%) with a diagnosis of probable iCAA, all of whom had received Lyodura material. Among these iCAA cases, three were 3 male (75%). All iCAA patients (100%) were operated on due to TBI. The disease course and neurological symptoms leading to the presentation of all four cases are provided in the Supporting Information (Tables 1 and S2). The median time from operation to onset or concurrent to iCAA diagnosis was 37.5 years (interquartile range [IQR] 35.8–46.0, range 35.0–46.0 years) (Tables 1 and 2). One non‐iCAA patient (1/45 [2%]) died 7 years after operation due to intracerebral hemorrhage; this person had multiple operations due to cerebral angioma; therefore, it was a non‐iCAA related death. Two patients with iCAA died: one from a traumatic intracerebral hemorrhage and the other from a spontaneous intracerebral hemorrhage. The cumulative risk for developing iCAA in recipients of any dura material was 5% (95% CI: 2–13), and 12% (95% CI: 4–28) for Lyodura recipients (Figure 2).

**TABLE 2:** Comparison of clinical characteristics between patients receiving some sort of dura material reconstruction and those without in pediatric patients undergoing neurosurgical procedures at the Christian‐Doppler‐University Hospital, Salzburg, Austria, period 1970–1996.

Characteristic	Dural substitute recipient *N* = 83^1^	Non‐recipient *N* = 305^1^
Men	63 (76)	205 (67)
Age at operation	13.9 (10.4–16.9)	12.0 (6.0–16.4)
Diagnosis at admission		
Traumatic brain injury	67 (81)	211 (69)
Intracranial tumor	12 (14)	34 (11)
Other	1 (1.2)	41 (13)
Intracranial hemorrhage	1 (1.2)	10 (3.3)
Intracranial abscess	1 (1.2)	6 (2.0)
Subarachnoidal bleeding	0 (0)	3 (1.0)
Unknown	1 (1.2)	0 (0)
Year of operation	1985 (1980–1988)	1982 (1974–1985)
Type of dura material used		
None	0 (0)	305 (100)
Unknown material	49 (59)	0 (0)
Lyodura	34 (41)	0 (0)
Years of follow‐up	37 (32–43)	41 (37–47)
Died	13 (16)	32 (10)
Age at death (years)	31 (25–43)	28 (18–36)
Cause of death		
Other	1 (7.7)	7 (22)
Intracranial tumor	2 (15)	5 (16)
TBI	2 (15)	4 (13)
Trauma other than TBI	2 (15)	3 (9.4)
Unknown	2 (15)	2 (6.3)
Intracranial hemorrhage	2 (15)	1 (3.1)
Cancer	0 (0)	3 (9.4)
Decompensatio cordis/myocardial infarction	2 (15)	2 (6.3)
Intracranial abscess	0 (0)	1 (3.1)
Hydrocephalus	0 (0)	1 (3.1)
Chronic lung disease	0 (0)	1 (3.1)
Pneumonia	0 (0)	1 (3.1)
Sepsis	0 (0)	1 (3.1)
CT available	15 (18)	18 (5.9)
MRI available	7 (8.4)	13 (4.3)
Diagnosis of iatrogenic CAA		
Not reported	68 (77)	270 (87)
None	16 (18)	41 (13)
Yes^a^	4 (5)	0 (0)
CAA diagnosis grade^b^		
Definite	2 (50)	0 (0)
Probable	1 (25)	0 (0)
Probable with supportive histology	1 (25)	0 (0)
Latency time in years	37 (36–41)	0 (0)
Diagnosis of stroke	0 (0)	4 (1)

*Note:* There were no deaths due to ischemic stroke recorded in the cohort.

Abbreviations: CAA, cerebral amyloid angiopathy; CT, computerized tomography of brain; MRI, magnetic resonance imaging of brain; TBI, traumatic brain injury.

^a^ All probable iatrogenic CAA.

^b^ According to Boston Criteria 2.0, or Edinburgh Criteria.

The occurrence of iCAA was significantly higher in patients who received some form of dura substitute *N* = 83 compared to those with only neurosurgery *N* = 305: 4/83 (1%) versus 0/305 (0%): OR 19 (95% CI 2–903) and *p* = 0.0022. Additionally, iCAA was more prevalent in patients who received Lyodura *N* = 34 (9%) compared to those who received dura material of unknown type or neurosurgery alone *N* = 354 (91%): *N* = 4 (11%) versus *N* = 0 (0%), OR 56 (95% CI 6–2667) and *p* < 0.001. (Table 3).

**TABLE 3:** Cumulative incidence of iCAA and stroke in pediatric patients treated with neurosurgical procedures at the Christian‐Doppler‐Klinik, Salzburg, Austria, period 1970–1996.

*N* = 388	iCAA+	iCAA−	OR (95% CI)	Ischemic stroke+	Ischemic stroke−	OR (95% CI)
Dura material+	4	79	18.9 (2.1–902.8)^a^	0	83	0.7 (0–7)^b^
Dura material−	0	305		4	301	
Lyodura+	4	30	55.8 (6–2667)^c^	0	34	2.0 (0.0–19)^d^
Lyodura−	0	354		4	350	
Dura only: *N* = 83						
Lyodura+	4	30	7.9 (1–388)^e^	0	34	1.4 (0–114)^b^
Lyodura−	0	49		0	49	

*Note:* Lyodura: usage of cadaveric dura material of Lyodura brand (+ recorded usage; − no recorded usage); Dura only: group of patients receiving any type of dura material; Fisher's exact test with odds ratio and continuity correction was used.

Abbreviations: − no signs of iCAA); CI, confidence interval; iCAA, iatrogenic cerebral amyloid angiopathy (+ confirmed as probably iCAA; OR, odds ratio.

^a^ *p* = 0.0022.

^b^ *p* = 1.0.

^c^ *p* < 0.001.

^d^ *p* = 0.444.

^e^ *p* = 0.078.

There were no patients that received dura material who developed a stroke later in life, whereas 4 without dura material did (OR 1 [95% CI 0–7] and *p* = 1.0, and OR 2 [95% CI 0–99.3] and *p* = 1.0) The Kaplan–Meier analysis indicates a significantly more frequent occurrence of CAA in Lyodura recipients at *p* < 0.001 (Figure 2).

## 4 Discussion

The cumulative incidence of iatrogenic cerebral amyloid angiopathy (iCAA) in the local pediatric population undergoing neurosurgery in 1970–1996 was approximately 1%. Among patients with documented Lyodura use, the incidence increased to 12%. The overall use of cadaveric dura materials (Lyodura) was 34 (9%) and dural grafts were placed in 83 (21%) procedures. The odds of developing iCAA after receiving a (cadaveric) dural graft were 20–50 times higher than after receiving a non‐(cadaveric) graft.

The estimated cumulative incidence contrasts sharply with iCJD, which had a lower incidence of 0.048% [11]. Both iCJD and iCAA can be transmitted through cadaveric dura material or human growth hormone, although other routes, such as hematogenous or through neurosurgical equipment, are possible [12]. Our finding is striking because our study might underestimate the true number of iCAA cases due to the high unavailability of neuroimaging (84%). The underestimation arises because we calculated the number of iCAA cases as a proportion of all cases available for review. Patients may lack neuroimaging for several reasons: they may not have developed iCAA yet due to the follow‐up time, in which case our estimation is accurate; they may have developed iCAA, but the symptoms were mild and not clinically noticeable, leading to underestimation; or they may have developed iCAA but sought treatment outside our institution, also resulting in underestimation. Notably, besides the four identified iCAA cases, there were no patients who had developed sporadic CAA (under generous assumptions mentioned above) and none had developed ischemic stroke after exposure to dural grafts, nor were there any in the non‐exposed group. These findings from the negative control group support the idea that cadaveric dura is an important risk factor for developing CAA after neurosurgical procedures. At the same time, although we collected the cause of death for the entire cohort and thereby ruled out iCAA‐related deaths in deceased patients, we most likely underestimated the number of patients with CAA in both the cadaveric dura group and those without. This is because deceased patients with minimal clinical manifestations (such as mild cognitive impairment or TFNE) would not have been identified in the ICD‐9/10 codes. This could have led to an overestimation of the observed effect. However, our cohort was relatively young at the time of censoring, making the occurrence of sporadic CAA unlikely.

The total population at risk of developing iCAA due to the use of Lyodura in the past might be relatively limited. Based on our study, approximately 12% of the patients exposed to cadaveric dura might develop iCAA. The total number of grafts used varies significantly by country: more than 2200 grafts have been utilized in Australia, fewer than 400 in the United States, and approximately 20,000 grafts annually over a 13‐year period in Japan [13, 14]. Although local differences in graft use might exist, our study provides a reference value for European countries, as few estimates of cadaveric graft use are available. Conservatively extrapolating our findings, perhaps < 250 persons per country might be at risk of developing iCAA.

We identified Lyodura as an important risk factor for iCAA and speculate that it might be a sufficient cause. In a previous study that investigated records from a large hospital, 8 out of 18 (44%) iCAA cases had confirmed exposure to cadaveric dura material in their past history [7]. An international iCAA case series, which included three of the cases presented here, found cadaveric material exposure in 8 out of 27 (30%) patients [6]. There is strong evidence supporting prion‐like transmission in cases involving human cadaveric gonadotropic hormone. Similarly, circumstantial evidence suggests that cadaveric dura may also play a role, as it can contain traces of amyloid‐beta (Aß), as demonstrated in previous studies [3, 15]. Furthermore, iatrogenic Creutzfeldt‐Jakob disease (iCJD) cases linked to cadaveric dura grafts have been shown to harbor Aß pathology [15, 16, 17]. While prion scrapie protein (PrP^sc^) and Aß deposition can occur concurrently, studies indicate that Aß can also be transmitted independently of PrP^sc^ [18]. At the same time, other routes of transmission are possible, although we could not find direct evidence for this in our cohort. Notably, all cases of iCAA in our cohort (4/4) had a history of traumatic brain injury (TBI), which warrants consideration as a potential contributing factor. TBI has been associated with altered beta‐amyloid metabolism and clearance, which could predispose individuals to CAA, particularly when compounded by neurosurgical interventions. This aligns with the ‘double‐hit’ hypothesis, where TBI initiates amyloid pathology, and subsequent neurosurgical exposure may amplify this effect. Literature on TBI has documented its role in the deposition of amyloid‐beta plaques and the disruption of glymphatic clearance pathways, mechanisms that are integral to the pathogenesis of CAA [19, 20, 21]. Therefore, the interaction between TBI and neurosurgical treatment could be a plausible pathway for the development of iCAA, emphasizing the need for further studies to explore this relationship in more detail.

To enhance case ascertainment and physician awareness, a surveillance system similar to that implemented for iCJD in Japan could be considered in countries where patients were most exposed to Lyodura [22]. The recent establishment of the international registry for iCAA plays a pivotal role in setting up this surveillance system [23].

## 5 Limitations and Strengths

Strengths of our study include the availability of detailed historical records dating back more than 50 years and complete coverage regarding the cause of death. This allowed us to estimate the number of patients who received Lyodura and the percentage of those who later developed iCAA. Further, the inclusion of a negative control group allowed a more robust estimation of cadaveric dura as a cause for developing CAA. Finally, we were able to accurately collect information about the exposures and diagnoses, which reduced measurement error in our estimates.

Our study has limitations. The accurate ascertainment of the cause of death, including potentially missed iCAA‐related deaths, depends on the absence of misclassification in ICD9/10 codes and the absence of linkage error between national statistics and local datasets. Such errors would lead to an underestimation of cases. As no registry of recipients of cadaveric or other types of dura grafts was available at the time of the study at our facility, we might have missed an unknown number of exposed patients. Second, the limited availability of direct follow‐up required suppletion of the mortality and cause of death data with national registry data. The majority of cases (91%) did not undergo specific diagnostic procedures to detect possible CAA (MRI data available for 5% of patients). However, this suppletion could have led to differential misclassification, particularly in persons with subclinical CAA or mild symptoms (mild cognitive impairment, TFNE). Third, we limited the inclusion criteria to patients aged 0–18 years to avoid overlap with sporadic CAA, which typically affects older adults, and because data on older patients were not available. Fourth, our study was not designed to address other modes of transmission, such as blood products [24]. Finally, the exact source of Lyodura could not be determined, which limits our ability to study whether grafts in patients who developed iCAA might have come from deceased individuals with substantial amounts of amyloid protein.

## 6 Conclusions

Our findings add to the evidence that cadaveric dura material, particularly the Lyodura brand, is a strong risk factor for developing probable iatrogenic cerebral amyloid angiopathy after a substantial latency period. The incidence surpasses that of iCJD by orders of magnitude. However, since not all exposed individuals developed the illness, further large‐scale susceptibility studies, similar to those conducted for the variant form of CJD, should be pursued.

## Author Contributions

**Slaven Pikija:** conceptualization; methodology; software; data curation; supervision; formal analysis; writing – original draft; investigation; validation. **Andreea Toma:** conceptualization. **Richard Radlberger:** data curation. **Christoph J. Griessenauer:** conceptualization. **Constantin Hecker:** conceptualization; data curation. **Eugen Trinka:** conceptualization; writing – review and editing. **Park Hyon Ki:** data curation. **Theo Kraus:** data curation. **Serge Weis:** data curation; investigation. **Marieke J. H. Wermer:** writing – review and editing. **Ellis S. van Etten:** writing – review and editing. **Kanishk Kaushik:** writing – review and editing; conceptualization.

## Conflicts of Interest

The authors declare no conflicts of interest.

## Supporting information


Data S1.

Data S2.

## Data Availability

The data that support the findings of this study are available from the corresponding author upon reasonable request.
