# Supplementary material for: Iatrogenic Cerebral Amyloid Angiopathy in Patients Treated With Cadaveric Dura Mater During Childhood Neurosurgery: A Retrospective Cohort Study
Source: Eur J Neurol. 2025 Mar 6;32(3):e70091. doi: 10.1111/ene.70091 (PMC11883419; doi:10.1111/ene.70091)
Supplement: Supplementary file 2 — Data S2. [file ENE-32-e70091-s002.docx]

**Iatrogenic cerebral amyloid angiopathy epidemiology**

**Supplemental Material**

**Estimates for the cohort with available modern medical records**

Among the patients successfully followed up N=61/388 (16%), additional diseases during the observation period included epilepsy N=21 (37%), ischemic stroke N=4 (7%), dementia N=1 (2%), medulloblastoma N=1 (2%), TBI N=1 (2%). Recipients of Lyodura N=11 (18%) did not have significantly more occurrence of epilepsy vs. those with neurosurgery alone or recipients of another dura substitute N=50 (82%): N=4 (36%) vs. N=15 (25%), OR 0.75, 95% CI 0.2-4.1, p=0.726. Among the patients receiving some sort of dura substitute N=20 (33%), compared to those who did not N=41 (67%), the occurrence of iCAA was significantly higher: N=4 (20%) vs. N=0 (0%), OR 12 (95% CI 1-596) and p=0.015. Among those receiving any type of dura substitute, the patients receiving Lyodura N=11 (18%), vs. those treated with other dura substitute or neurosurgery alone N=50 (82%), the occurrence of iCAA was significantly higher: N=4 (36%), vs. N=0 (0%): OR 29 (9% CI 3-1519) and p<0.001. There were no differences in ischemic stroke occurrence between patients receiving dura material or not (Supplemental Table 2).

**Methodology used by Statistik Austria for searching national population registry.**

Since it is unreasonable to question relatives about causes of death immediately after a death occurs, the Research Organization Act provides for the possibility of transferring mortality data (date of death and cause of death) through the Austrian Federal Institute of Statistics (Statistics Austria) solely for medical-scientific purposes.

According to Section 2f of the Research Organization Act, mortality matching is conducted exclusively for medical-scientific institutions that can present a positive ethics committee decision. The positive ethics committee decision must be attached to the agreement.

Matching can be performed for the period from 1970 to the most recent reporting year. As a standard result, the date of death and cause of death (ICD code) are transmitted.

For the matching, the following fields must be present in this order: last name, first name, birth day, birth month, birth year, gender. Names with special characters should be converted to a simplified form where possible (e.g., René as Rene). Additional fields can also be appended to the end of the file. These will be ignored during matching but will be output unchanged in the result file.

An exact match of birth date and gender is required for a successful data match. In the first program run, the software searches for a complete match of all characteristics. If no match is found, the name fields are phonetically transformed (e.g., umlauts simplified, double letters reduced, etc.). Double names are also separated, and each name part is searched individually for matches. The search ends at the first match. After applying these simplifications, all personal characteristics must match to be identified as a match. Partial matches are not allowed for data protection reasons. The program does not recognize typographical errors.

**De-identified clinical data on four cases of iatrogenic cerebral amyloid angiopathy**

Case Vignettes

Patient 1:

A 43-year-old male patient with a history of traumatic brain injury as an infant, treated with osteoplastic trepanation and palacos osteoplasty. At the age of 36, he presented with an acute atypical bilateral parieto-temporal intracranial hemorrhage, followed by another multifocal intracerebral hemorrhage. A diagnostic biopsy of leptomeningeal and cortical tissue confirmed amyloid angiopathy with beta-amyloid plaques. PET-CT imaging showed amyloid deposits in the frontal, parietal, temporal cortex, and praecuneus. No signs of vasculitis, aneurysm, or arteriovenous malformation were detected on pan-angiography. MRIs from 2014 onward demonstrated bi-hemispheric cerebral superficial hemosiderosis and microbleeds. Genetic testing for APP, CST3, ITM2B, and TTR revealed no mutations.

Patient 2:

This patient was diagnosed with cerebral amyloid angiopathy (CAA) post-mortem. At the age of 8, the patient suffered a traumatic brain injury, leading to epidural hematoma evacuation and duraplasty. He died at age 43 following a moped accident that caused severe brain trauma and intracranial hemorrhage. Post-mortem autopsy confirmed the presence of beta-amyloid plaques and CAA. Genetic tests for APP, CST3, ITM2B, and TTR were negative.

Patient 3:

A 45-year-old female patient with a history of three intracerebral hemorrhages. At the age of 8, she suffered a left frontal fracture from a fall and underwent surgery involving lyophilized dura. At 43, she experienced her first hemorrhage in the left hemisphere, followed by another in the right occipital region. Despite interventions, including decompressive craniotomy and ventricular drainage, she died from brainstem compression and cerebral hemorrhage. Autopsy revealed extensive beta-amyloid deposits consistent with CAA. Genetic testing for APP, CST3, ITM2B, and TTR showed no mutations.

Patient 4:

A 53-year-old male with a history of a severe traumatic brain injury at age of 7, followed by symptomatic epilepsy. He presented with left frontal intracerebral hemorrhage and right-sided weakness. Imaging revealed atypical hemorrhage but no signs of aneurysm or arteriovenous malformation. Genetic testing for APP, CST3, ITM2B, and TTR showed no mutations. He experienced multiple complications, including a secondary hydrocephalus and seizures, requiring external ventricular drainage and intensive neurorehabilitation.

Supplemental Table 1. Cumulative incidence of iCAA and stroke in pediatric patients treated with neurosurgical procedures at the Christian-Doppler-Klinik, Salzburg, Austria, period 1970-1996.

|  | iCAA + | iCAA - | OR (95% CI) |  | Ischemic Stroke + | Ischemic  Stroke - | OR (95% CI) |
| --- | --- | --- | --- | --- | --- | --- | --- |
| N=61 |  |  |  |  |  |  |  |
| Dura material + | 4 | 16 | 11.8 (1-596)^#^ |  | 0 | 20 | 0.4 (0.0-4)^£^ |
| Dura material - | 41 | 0 |  |  | 4 | 37 |  |
|  |  |  |  |  |  |  |  |
| Lyodura + | 4 | 7 | 29.2 (3-1519)^ß^ |  | 0 | 0 | - |
| Lyodura - | 0 | 50 |  |  | 4 | 57 |  |

Cohort 2: patient with available modern medical records; Lyodura: usage of cadaveric dura material of Lyodura brand (+ recorded usage; - no recorded usage); Dura only: group of patients receiving any type of dura material; iCAA: iatrogenic cerebral amyloid angiopathy (+ confirmed as probably iCAA; - no signs of iCAA); OR – odds ratio; CI – confidence interval. Fisher´s exact test with odds ratio and continuity correction was used.

& p = 0.08; # p = 0.0146; ß p<0.001; £ p=0.655

**Supplemental Table 2**. Comparison of clinical characteristics between patients developing iatrogenic cerebral amyloid angiopathy and those without in pediatric patients undergoing neurosurgical procedures at the Christian-Doppler-Klinik, Salzburg, Austria, period 1970-1996.

| **Characteristic** | iCAA, N = 4*^1^* | Without iCAA, N = 384*^1^* |
| --- | --- | --- |
| Man | 3 (75) | 265 (69) |
| Age at operation | 6.5 (4.2 – 7.9) | 12.6 (6.6 – 16.6) |
| Diagnosis at admission |  |  |
| Traumatic brain injury | 4 (100) | 274 (71) |
| Intracranial tumor | 0 (0) | 46 (12) |
| Other | 0 (0) | 42 (11) |
| Intracranial hemorhage | 0 (0) | 11 (2.9) |
| Intracranial abscess | 0 (0) | 7 (1.8) |
| Subarachnoidal bleeding | 0 (0) | 3 (0.8) |
| Unknown | 0 (0) | 1 (0.3) |
| Year of operation (IQR, range) | 1978 (1976 – 1980, 1974-1984) | 1983 (1976 – 1986, 1970-1996) |
| Type of dura material used |  |  |
| None | 0 (0) | 305 (79) |
| unknown material | 0 (0) | 49 (13) |
| Lyodura | 4 (100) | 30 (7.8) |
| Follow-up in years | 38 (36 – 41) | 41 (37 – 47) |
| Died during follow-up | 2 (50) | 43 (11) |
| Age at death (years) | 44 (44 – 45) | 29 (18 – 36) |
| Cause of death |  |  |
| Other | 0 (0) | 8 (19) |
| Intracranial tumor | 0 (0) | 7 (16) |
| TBI | 0 (0) | 6 (14) |
| Trauma other than TBI | 0 (0) | 5 (12) |
| Unknown | 0 (0) | 4 (9.3) |
| Intracranial hemorhage | 2 (100) | 1 (2.3) |
| Cancer | 0 (0) | 3 (7.0) |
| Heart failure | 0 (0) | 2 (4.7) |
| Heart infarction | 0 (0) | 2 (4.7) |
| Intracranial abscess | 0 (0) | 1 (2.3) |
| Hydrocephalus | 0 (0) | 1 (2.3) |
| Chronic lung disease | 0 (0) | 1 (2.3) |
| Pneumonia | 0 (0) | 1 (2.3) |
| Sepsis | 0 (0) | 1 (2.3) |
| CT available | 3 (75) | 30 (7.8) |
| MRI available | 1 (25) | 19 (4.9) |
| CAA diagnosis grade |  |  |
| Definite | 2 (50) | 0 (0) |
| Probable | 1 (25) | 0 (0) |
| probable with supportive histology | 1 (25) | 0 (0) |
| Latency time in years | 38 (36 – 41) | - |
| Diagnosis of stroke | 0 (0) | 4 (1) |

TBI – traumatic brain injury; CAA – cerebral amyloid angiopathy; CT – computerized tomography of brain; MRI – magnetic resonance imaging of brain
